# Assessing the acceptability and implementation feasibility of a culturally adapted parenting intervention for Marshallese mothers: A study protocol

**DOI:** 10.1016/j.conctc.2023.101240

**Published:** 2023-12-12

**Authors:** Eliza Short, Sarah K. Council, Ashlea Bennett Milburn, Alice Ammerman, Jennifer Callaghan-Koru, Philmar Mendoza Kabua, Britni L. Ayers

**Affiliations:** a University of Arkansas for Medical Sciences Northwest, 2708 S. 48th St., Springdale, AR, 72762, USA; b University of Arkansas, 1 University of Arkansas, Fayetteville, AR, 72701, USA; c University of North Carolina at Chapel Hill, 1700 MLK, Chapel Hill, NC, 27599, USA

**Keywords:** Culturally-adapted intervention, Pacific Islander, Marshallese, Childhood obesity, Centering Parenting

## Abstract

**Background:**

Pacific Islanders, including the Marshallese, face higher rates of obesity and obesity-related chronic conditions. Early-life interventions targeting eating patterns during the first 1000 days of life are essential to promote proper nutrition and growth. Marshallese mothers and caregivers are important decision-makers for feeding practices that could affect childhood obesity rates in Marshallese children. However, little is known about dietary patterns and practices of Marshallese families from birth to 12 months. Culturally-adapted approaches using community-based assets and Pacific Islander cultural values/practices have demonstrated effectiveness in reducing obesity but have not been developed for children.

**Methods:**

This article describes the protocol for a study to culturally adapt the Centering Parenting intervention for Marshallese mothers in Arkansas.

**Conclusion:**

This will be the first study to culturally adapt and implement Centering Parenting with Marshallese women in the United States. This study will be an important first step to assess the feasibility and acceptability of an abbreviated parenting intervention to reduce childhood obesity in Marshallese communities.

## 1 Introduction

Pacific Islanders face high rates of obesity and obesity-related chronic conditions [[1], [2], [3], [4], [5], [6], [7], [8], [9], [10], [11], [12], [13], [14], [15], [16]]. The Centers for Disease Control and Prevention’s (CDC) National Health Interview Survey documented that 53.1% of Pacific Islanders surveyed in the United States (US) in 2016 were obese, compared to 39.6% of African Americans and 38.9% of American Indian or Alaska Natives [17]. The health-related indicators of Pacific Islanders residing in northwest Arkansas are similarly concerning. Our preliminary study with Marshallese in Arkansas revealed that 41.2% of participants had measures indicating hypertension, 16.4% had prehypertension, and 90% were overweight or obese (*n* = 401). Additionally, diabetes screenings indicated that 38.4% of participants had HbA1c levels indicative of diabetes, and an additional 32.6% had levels indicative of prediabetes [18].

Early-life interventions targeting eating patterns during the first 1000 days of life are essential to promote proper nutrition and growth and reduce the risk of childhood obesity [[19], [20], [21]]. The World Health Organization and the American Academy of Pediatrics recommend exclusive breastfeeding for six months, with introduction of appropriate complementary foods after six months [22,23]. The introduction of solid foods prior to four months is associated with obesity later in life, while exclusive breastfeeding for at least six months can reduce the odds of becoming overweight by more than 30%, as breast milk’s protective effect increases with duration [24,25].

Exclusive breastfeeding rates among Marshallese in the US are below desired levels [[26], [27], [28]]. A national survey of Marshallese mothers in the US found that only 31% were exclusively breastfeeding their infants at four months of age [29]. Studies in Arkansas have demonstrated fewer than 2% of Marshallese mothers were exclusively breastfeeding their infants at six months of age, and the mothers reported experiencing numerous structural and socio-cultural barriers to exclusive breastfeeding [[26], [27], [28]]. In addition to Marshallese infants receiving introduction of foods other than breastmilk earlier than at six months of age, the foods being introduced are high in simple carbohydrates, typically including rice, starchy fruits, juice, and pureed fruit with canned milk [[30], [31], [32]]. Meanwhile, the Dietary Guidelines for Americans 2020–2025 recommends the introduction of a variety of nutrient-rich foods, such as fruits, vegetables, whole grains, and lean proteins, while limiting refined grains, added sugars, and foods high in sodium [20].

Marshallese mothers and caregivers are important decision-makers for feeding practices within the household, by planning and preparing meals for the family [32]. Qualitative research has demonstrated the value of collectivist culture among the Marshallese community, where families eat together from “one pot” [33]. However, little is known about the dietary patterns and practices of Marshallese families that include infants up to 12 months of age. Current individual-level parental interventions addressing obesity are often not culturally tailored and may not be effective for the Marshallese population where traditional diets and collectivist approaches (value group over individual) are more common/valued [34]. There is a need for a culturally tailored parenting intervention that focuses on dietary patterns and practices specific to the Marshallese community, to effectively reduce the prevalence of childhood obesity [32].

“Centering Parenting” is a group-model parenting intervention that has demonstrated effectiveness in improving exclusive breastfeeding, appropriate introduction of complementary foods, and healthy dietary patterns [[35], [36], [37]]. Culturally adapted approaches using community-based assets and Pacific Islander cultural values/practices have demonstrated effective in significantly improving obesity, but have not focused on childhood obesity [38].

The purpose of this study is to culturally adapt a group-model parenting intervention for Marshallese mothers of newborns focused on the nutritional health of children from birth to 12 months, including exclusive breastfeeding, appropriate introduction of complementary foods, and healthy dietary practices among both mothers and children. We will collect pre-intervention dietary recall data from Marshallese mothers of young children, providing a rich characterization of the dietary patterns of the target population; use these data and a culturally grounded theory approach to adapt Centering Parenting for Marshallese mothers; and assess the acceptability and implementation feasibility of this tailored intervention using the criteria of cultural adaptation satisfaction and the Reach, Effectiveness, Adoption, Implementation, and Maintenance (RE-AIM) framework [39]. This article describes the design and protocol for this cultural adaptation study.

## 2 Methods and analysis

This study is approved by the University of Arkansas for Medical Sciences Institutional Review Board (#274752).

### 2.1 Study aims

The goal of this study is to culturally adapt the Centering Parenting intervention for the Marshallese community in Northwest Arkansas. The protocol for this empirical adaptation includes three phases/aims: 1) conduct three 24-h dietary recall and infant feeding style surveys with 20 Marshallese mothers with infants under 12 months using the Nutrition Data System for Research software to characterize dietary patterns and practices of Marshallese mothers of young children; 2) use a culturally-grounded theory approach [40,41], Marshallese stakeholder input, and the data from Aim 1 to adapt Centering Parenting to the cultural preferences of Marshallese women and their families, with a focus on exclusive breastfeeding, appropriate introduction of complementary foods, and feeding behaviors aimed at preventing obesity; and 3) assess the acceptability and implementation feasibility of an abbreviated version of the culturally adapted intervention, Centering Parenting, using criteria for cultural adaptation satisfaction and implementation science principles guided by the RE-AIM framework [39].

### 2.2 Approach

This study will use a culturally grounded approach to culturally adapt the Centering Parenting intervention using a Marshallese community advisory board (CAB) and Marshallese stakeholder input [40,41]. Culturally grounded approaches utilize methods that place the culture and social context of the targeted population at the center of the intervention [40,41]. Methods are used in which curriculum components evolve from the ground up (i.e., from the worldviews, values, beliefs, and behaviors of the population that the program is intended to serve) [40,41]. While time and cost investments are high, they may be necessary for unique populations in which available evidence-based practices have limited applicability or generalizability. Culturally grounded approaches to adapt evidence-based interventions have been demonstrated as highly effective with Native Hawaiian and Pacific Islander populations [[38], [42], [43]].

Additionally, a Community-Engaged Research (CER) approach will be used in the design and implementation of this study. CER is an approach to honor and integrate Marshallese cultural values and practices into every aspect of the research [[44], [45], [46], [47], [48], [49], [50], [51]]. A CER approach allows community knowledge to inform all aspects of the research to be more culturally-acceptable and has demonstrated effectiveness in building alliances to improve health when disparities result from systematic disadvantage, racism, and historical trauma [[44], [45], [46], [47], [48], [49], [50], [51]]. To ensure cultural appropriateness, this study is guided by the Healthy Start Community Action Network (CAN) that includes local health care professionals, Marshallese community members, and an interprofessional research team. The interprofessional research team includes quantitative and qualitative researchers, as well as Marshallese bilingual study staff to provide feedback of study materials and input on how to modify the study materials and protocol to be culturally-appropriate for Marshallese participants [52].

### 2.3 Study design

A mixed methods design will be used to culturally adapt the Centering Parenting intervention for Marshallese mothers in Arkansas. A multi-methods design was chosen to ensure a more comprehensive view of participants’ acceptability and implementation feasibility of the cultural adaptation of Centering Parenting. In the first phase, we will collect pre-intervention 24-h dietary recall data from Marshallese mothers of young children providing a rich characterization of the dietary patterns of the target population; use these data and a culturally grounded theory approach to adapt Centering Parenting for Marshallese mothers; and assess the acceptability and implementation feasibility of this tailored intervention using the criteria of cultural adaptation satisfaction and the RE-AIM framework.

### 2.4 Centering Parenting intervention overview

Centering Parenting is an evidence-based intervention that challenges the standard model of individual-level parenting interventions [37]. The intervention occurs over nine group sessions from six weeks through 12 months of the newborn’s life. Group visits are 90–120 min each and follow a unique structured curriculum that incorporates standards of care around parenting, infant feeding practices, and nutrition. Further, this type of group-model intervention has demonstrated effective in improving exclusive breastfeeding and delayed introduction of complementary foods in other populations [[35], [36], [37],^53^]. In the Centering sessions, credentialed providers offer: 1) brief one-on-one exams; 2) facilitated discussion around parenting topics; and 3) a 10–15 min closing for group questions.

Following cultural adaptation, we will use the RE-AIM Planning Tool developed by Glasgow and colleagues to consider various aspects of the intervention that will have implications for the success of a future effectiveness trial [39]. This will include elements of the framework such as participant recruitment strategies to reach the target population, potential adoption by the host or similar organizations delivering the program, whether it can be implemented by staff in other organizations, and potential for maintaining the program given available resources and staff [39].

For this study, we will implement an abbreviated version of the culturally adapted intervention with Marshallese mothers to assess the acceptability and implementation feasibility prior to implementing the full intervention. The abbreviated culturally adapted intervention (3 sessions specific to infant feeding/nutrition) will be piloted at the Jones Center, a community-based organization, by an Assistant Clinical Professor in the Department of Family and Preventive Medicine, and a bilingual Marshallese Registered Nurse. Both are trained in Centering facilitation. Centering Parenting participants may bring their infants.

### 2.5 Recruitment, consent, and retention

The study will include a total of 20 Marshallese women with children under 12 months of age to participate in all three phases of the study. Recruitment will take place via trained bilingual research staff. Potential participants will be recruited from our Healthy Start program that enrolls approximately 300 Marshallese women a year making this recruitment goal highly attainable. Eligibility criteria includes being female, being ≥18 years, being self-reported Marshallese, and having children under 12 months of age. Potential participants who meet the eligibility criteria will be asked if they would like to participate and be recruited by trained bilingual Marshallese community health workers and through our relationships with community organizations, specifically the Marshallese Education Initiative, Arkansas Coalition of Marshallese, Marshallese pastors, and other community contacts developed through prior Community-Based Participatory Research Program fieldwork. We have developed flyers with Marshallese stakeholders to place in the Family Medicine Clinic and among our Healthy Start Cohort.

Potential participants who meet the eligibility criteria will be offered the opportunity to join the study and complete the consent process prior to the first data collection event. Trained bilingual female Marshallese study staff will conduct the consent process. The bilingual study staff will read the consent aloud to the participants in the participant’s language of choice (English or Marshallese). Participants will be given the opportunity to have their questions answered prior to consent. Each participant will be provided a copy of the consent in either/both English and Marshallese.

The CER team will use an engaged approach to collaboratively develop a retention plan with Marshallese stakeholders. The retention plan specifies that all study staff responsible for retention will be bilingual (Marshallese/English). Marshallese bilingual study staff will obtain each participant’s contact information and preferred method of contact. Marshallese bilingual study staff will also collect contact information for at least two relatives and ask participants for permission to contact their relatives if needed. Confidentiality rules will be followed, and no participant information will be provided to relatives. Before each data collection visit, bilingual study staff will contact study participants about the upcoming data collection visit. If a participant withdraws, the study team will document who withdrew and why they withdrew. Each Marshallese participant will receive a $20 gift card upon completion of each 24-hr dietary recall data collection. Each Marshallese participant will receive a $50 gift card if they participate in both the survey and focus group session.

### 2.6 Data collection

Study procedures (e.g., consent process, focus groups, surveys, data collection, administering compensation) will be conducted in person or remotely. In either case, tools such as phone, video, email, text, and online data collection tools (e.g., REDCap) may be used [54]. Dietary recall data collection will take up to 30 min per recall, and the focus group and survey will last about 2 h total.

#### 2.6.1 Phase 1: dietary recall

Twenty (n = 20) participants will participate in three dietary recalls prior to the adaptation and implementation. After the three sessions have been adapted and piloted, all participants will participate in both a survey and focus group. All data collection will be conducted by Marshallese community health workers.

Each participant will be invited to provide dietary intake information prior to the cultural adaptation using 3 24-h dietary recall interviews conducted over the phone. Dietary intake information will be applied to the Healthy Eating Index (HEI)-2015, which is a tool used to evaluate the extent to which dietary intake meets the Dietary Guidelines for Americans 2015–2020 [55]. The HEI-2015 consists of 13 separate nutrient components summed to create a total score (0–100), with higher scores representing higher diet quality. The HEI-2015 consists of nine adequacy components to emphasize in the diet (e.g., total fruits, total vegetables, whole grains) and four components to consume in moderation (e.g., added sugars, sodium).

#### 2.6.2 Phase 2: cultural adaptation

Participant input on the adaptation will be solicited through focused group discussion regarding the Centering Parenting and proposed changes from the study team.

#### 2.6.3 Phase 3: assessment of acceptability and feasibility

Acceptability of the cultural adaptation will be assessed using an adapted version of a Satisfaction with Brief Intervention Survey described below [56].

To assess implementation feasibility, we will apply principles of the RE-AIM planning framework and tool prior to implementation with our Centering Steering Committee members [57,58]. Applying the RE-AIM Planning Tool serves as a checklist for key issues that should be considered when planning an intervention (i.e., demographics of target population relative to the target audience, potential barriers to intervention delivery, etc.). This will guide the research team in a strategic approach to improve the internal and external validity of the study and inform a future effectiveness study [57,58]. Following the brief pilot, semi-structured interviews with intervention staff will provide insight on implementation feasibility including facilitators and barriers.

### 2.7 Instruments

The quantitative surveys and focus group guides were developed with extensive input from Marshallese stakeholders and are specific to this study. After the instruments were initially drafted with stakeholders, the CER team met monthly with two female Marshallese bilingual study staff who will be implementing the data collection in-language with the participants.

#### 2.7.1 Dietary assessment

The Nutrition Data System for Research (NDSR) software version 2022 will facilitate dietary recalls, which guides data collectors through a multiple-pass approach to collect complete dietary intake information [59]. NDSR includes more than 19,500 foods with values for 178 nutrients and food components that may be used to calculate HEI-2015 scores.

#### 2.7.2 Quantitative

Acceptability of the cultural adaptation will be assessed using an adapted version of a Satisfaction with Brief Intervention Survey [56]. The survey will consist of questions focused on cultural appropriateness and satisfaction of the cultural adaptation of Centering Parenting, as well as demographic characteristics of participants. The survey will have three domains: 1) attitude (i.e., how you feel about the abbreviated culturally adapted intervention); 2) burden (i.e., challenges of the brief intervention in terms of comprehension, class attendance etc.); and 3) perceived effectiveness (i.e., perceived usefulness of the abbreviated culturally adapted intervention).

#### 2.7.3 Qualitative

Acceptability will also be evaluated through semi-structured focused groups regarding cultural appropriateness and satisfaction of the cultural adaptation of the abbreviated version of Centering Parenting. The qualitative focus group guide includes a series of open-ended questions with structured probes to elicit information regarding cultural adaptation satisfaction. Focus groups were chosen as the method of data collection as Marshallese prefer group discussion and this has demonstrated as an effective method [34].

After qualitative and quantitative data collection instruments were confirmed by community stakeholders, Marshallese bilingual study staff translated the instruments into Marshallese. After translations were complete, the CER team met with the Marshallese bilingual study staff and conducted two mock data collection events over the course of three months. These mock data collection events served as training for the Marshallese bilingual study staff and allowed the team to evaluate any challenges in cultural nuance, comprehension, and translations. All data documents went through three iterations. When conducting CER, it is critical to allow for time and flexibility to guarantee the study and data instruments are culturally appropriate to the target community [60].

### 2.8 Setting

Dietary recall data collection will take place over the phone and will include three non-consecutive days of intake, including one weekend and two weekdays. Centering Parenting sessions and data collection will take place at the Jones Center, a community-based organization located in Springdale, Arkansas.

## 3 Data analysis

### 3.1 Dietary recall

The National Cancer Institute’s Simple HEI scoring algorithm will be used to derive ratios from NDSR data for each of the 13 dietary components as either a percentage of total energy intake or per 1000 calories, with the exception of fatty acids which will be expressed as a ratio of unsaturated fatty acids to saturated fatty acids. Dietary component ratios will be averaged across the three dietary recall records. Scoring standards will then be applied to calculate the 13 individual HEI-2015 component scores and summed to create an overall HEI-2015 score for each participant [61]. The average HEI-2015 component scores from the sample will be used to adapt the nutrition education provided within the Centering Parenting intervention, by comparing the average score to the maximum possible score in each component. For example, if the average total fruit component score is well below the maximum, nutrition education may be adapted to focus on culturally appropriate fruits to emphasize in the diet. Specific adaptations will also consider foods that are acceptable to the family and already being consumed within the home and, therefore, within the social and financial constraints of the target population. Prior qualitative research has found Marshallese communities' diets include high intakes of white rice, processed meats, and sugar sweetened beverages [62]. Adaptations may include focusing on smaller portion sizes of refined grains and processed meats and emphasizing intake of no-sugar added beverages. Individual food-level data from the dietary recalls will also be examined to understand the frequency of intake of specific NDSR food groupings comparable to the What We Eat in America (WWEIA) Food Categories (e.g., mixed dishes, grains, snacks and sweets) [63,64]. This analysis will provide a deeper understanding of food groupings consumed most often to further tailor nutrition education to focus on nutritious food choices from frequently consumed food categories.

### 3.2 Quantitative surveys

While the primary purpose of collecting quantitative data will be to support and triangulate the qualitative data, exploratory analyses of the quantitative data will be conducted. Quantitative data analyses of the survey results will use descriptive statistical methods (e.g., percentages, means, etc.) to summarize the three domains addressed in the quantitative survey: 1) attitude (i.e., how you feel about the abbreviated culturally adapted intervention); 2) burden (i.e., challenges of the brief intervention in terms of comprehension, class attendance, etc.); and 3) perceived effectiveness (i.e., perceived usefulness of the abbreviated culturally adapted intervention).

### 3.3 Qualitative

Qualitative data from the focus groups will be audio recorded and transcribed verbatim in the language it was spoken by a Marshallese bilingual study staff. Any information transcribed in Marshallese will then be translated into English. Three researchers with qualitative interview experience will start with initial coding, which consists of naming each data segment with short summations. This process helps organize the data for focused codes. The focused codes that emerge will be used to identify and develop the most salient categories within the data [65,66]. The research team will collaboratively discuss the themes in order to ensure scientific rigor and inter-coder agreement. There will be two primary coders and one confirmation coder. Then, utilizing standard qualitative analysis, an inductive process will be used to identify and code emerging themes [65,66]. The qualitative analytic approach will integrate inductive and deductive techniques, and the codebook will include *a priori* thematic codes that represent themes from the interview guide and emergent codes that capture unanticipated categories of analysis.

## 4 Strengths and limitations

This will be the first study to assess the acceptability and implementation feasibility of a culturally adapted parenting intervention for Marshallese mothers in the US. The study will only include women in Arkansas; therefore, the results of this study may not be generalizable to other Pacific Islanders residing outside of Arkansas.

## 5 Dissemination

Effective dissemination is crucial to achieving research impact and is a key component to conducting CER. The research team will use the Agency for Healthcare Research and Quality’s Dissemination Planning Tool as the framework for our dissemination [67]. Specifically, we will disseminate results to study participants, research stakeholders (clinics, faith-based organizations, CAN, and community-based organization), the broader Marshallese community, and fellow researchers. Results will be disseminated to study participants through a one-page summary that shows the aggregated research results using plain language and infographics. To extend the reach, this information will be reviewed in a town hall meeting and disseminated using social media. No individual participant information will be shared, and all confidentiality procedures will be maintained. The data will be published in peer-reviewed journal articles and presented at academic conferences. Results of this study may be used for presentations, posters, or publications. The publications will not contain any identifiable information that could be linked to a participant. Aggregated results may be returned to participants in an infographic that is provided in person, via e-mail, and/or mailed to the participants. We will also work with stakeholders to disseminated aggregated information in town hall meetings.

## 6 Summary

Pacific Islanders face higher rates of obesity and obesity-related chronic conditions [[1], [2], [3], [4], [5], [6], [7], [8], [9], [10], [11], [12], [13], [14], [15], [16]]. The health disparities of Pacific Islanders residing in northwest Arkansas are of similar concern [18]. Early-life interventions targeting eating patterns during the first 1000 days of life are essential to promote proper nutrition and growth and reduce the risk of childhood obesity [[19], [20], [21]]. Marshallese mothers and caregivers are important decision-makers for household feeding practices [32]. However, little is known about dietary patterns and practices of Marshallese families with newborns to 12 months. Culturally adapted approaches using community-based assets and Pacific Islander cultural values/practices have demonstrated effective in significantly improving obesity, but have not been focused on childhood obesity [38]. Marshallese are disproportionately burdened by poor maternal and infant health outcomes, and early and consistent prenatal care are a mitigating factor. This will be the first study to culturally adapt and implement Centering Parenting with Marshallese women in the US. This study will be an important first step to assess the feasibility and acceptability of an abbreviated parenting intervention to reduce childhood obesity in Marshallese communities. Next steps include testing the efficacy of the culturally adapted Centering Parenting intervention on dietary and anthropometric outcomes at scale. Furthermore, there is evidence that utilizing behavioral interventions validated in one Pacific Islander group can be effective in other Pacific Islander populations. Establishing the evidence-base for interventions designed for Pacific Islanders may also inform work with other disenfranchised and indigenous populations who have strong collectivist cultures, thus increasing the generalizability of the proposed research [[68], [69], [70], [71], [72]].

## CRediT authorship contribution statement

**Eliza Short:** Conceptualization, Writing – review & editing. **Sarah K. Council:** Project administration, Writing – review & editing. **Ashlea Bennett Milburn:** Writing – review & editing. **Alice Ammerman:** Writing – review & editing. **Jennifer Callaghan-Koru:** Writing – review & editing. **Philmar Mendoza Kabua:** Writing – review & editing. **Britni L. Ayers:** Conceptualization, Funding acquisition, Methodology, Project administration, Writing – original draft.

## Declaration of competing interest

The authors declare that they have no known competing financial interests or personal relationships that could have appeared to influence the work reported in this paper.
